# *Mycobacterium bovis* BCG infection severely delays *Trichuris muris* expulsion and co-infection suppresses immune responsiveness to both pathogens

**DOI:** 10.1186/1471-2180-14-9

**Published:** 2014-01-17

**Authors:** Hendrik J Nel, Nelita du Plessis, Leanie Kleynhans, André G Loxton, Paul D van Helden, Gerhard Walzl

**Affiliations:** 1 Division of Molecular Biology and Human Genetics, MRC Centre for Molecular and Cellular Biology, NRF/DST Centre of Excellence in Biomedical TB Research, Faculty Medicine and Health Sciences, Stellenbosch University, Tygerberg, South Africa; 2 University of Queensland Diamantina Institute, Brisbane, QLD, Australia

**Keywords:** Helminth, Co-infection, Mycobacteria, Tuberculosis

## Abstract

**Background:**

The global epidemiology of parasitic helminths and mycobacterial infections display extensive geographical overlap, especially in the rural and urban communities of developing countries. We investigated whether co-infection with the gastrointestinal tract-restricted helminth, *Trichuris muris*, and the intracellular bacterium, *Mycobacterium bovis (M. bovis)* BCG, would alter host immune responses to, or the pathological effect of, either infection.

**Results:**

We demonstrate that both pathogens are capable of negatively affecting local and systemic immune responses towards each other by modifying cytokine phenotypes and by inducing general immune suppression. *T. muris* infection influenced non-specific and pathogen-specific immunity to *M. bovis* BCG by down-regulating pulmonary TH1 and Treg responses and inducing systemic TH2 responses. However, co-infection did not alter mycobacterial multiplication or dissemination and host pulmonary histopathology remained unaffected compared to BCG-only infected mice. Interestingly, prior *M. bovis* BCG infection significantly delayed helminth clearance and increased intestinal crypt cell proliferation in BALB/c mice. This was accompanied by a significant reduction in systemic helminth-specific TH1 and TH2 cytokine responses and significantly reduced local TH1 and TH2 responses in comparison to *T. muris*-only infected mice.

**Conclusion:**

Our data demonstrate that co-infection with pathogens inducing opposing immune phenotypes, can have differential effects on compartmentalized host immune protection to either pathogen. In spite of local and systemic decreases in TH1 and increases in TH2 responses co-infected mice clear *M. bovis* BCG at the same rate as BCG only infected animals, whereas prior mycobacterial infection initiates prolonged worm infestation in parallel to decreased pathogen-specific TH2 cytokine production.

## Background

Tuberculosis (TB) is most prevalent in resource-poor countries and factors such as genetic susceptibility, malnutrition and circulating strain differences have been implicated as determinants of TB disease development in these regions [1,2]. Compelling evidence demonstrates that many of these factors increase disease risk partly though the induction of host immune dysregulation and ultimately affect host control of *Mycobacterium tuberculosis (M. tb)* proliferation [3]. The high prevalence of parasitic helminth infections in TB affected communities, has highlighted co-infection as another risk factor compromising host immunity and thus a potential determinant for development of TB [4,5]. In support of this theory, several reports indicated that TB patients are commonly found to be co-infected with helminth species such as *Trichuris trichiura* and *Ascaris lumbricoides*[6] and present with increased total and helminth-specific serum immunoglobulin E (IgE) [7].

Host control of mycobacterial or helminth infections largely rely on the induction of appropriately polarized immune responses. Protective immune responses to *M. tb* infection are associated with enhanced T helper 1 (TH1) type cellular immunity and the production of characteristic TH1 cytokines such as tumor necrosis factor alpha (TNF-α), interferon-gamma (IFN-γ) and interleukin-12 (IL-12) [8]. Conversely, protection against most helminths requires a T helper 2 (TH2) type cellular immune response with production of distinct TH2 cytokines such as IL-4, IL-5, IL-13 and IL-9 [9,10]. Since TH1 and TH2 immune responses have the ability to concurrently down-regulate each other, a state of co-infection could result in inappropriate protective host immune responses to either infections [11]. Furthermore, both pathogens have the potential to induce regulatory T cell (Treg) responses associated with production of immune suppressive cytokines such as IL-10 and transforming growth factor beta (TGF-β) [10-13].

In line with the TH1/TH2 dichotomy, hypotheses concerning helminth-mycobacterial co-infection postulate that a helminth-induced TH2 immune bias could inhibit development of protective cellular immune responses to *M. tb*, increase mycobacterial proliferation or lead to the failure of vaccine strategies against TB [14,15]. This theory is supported by numerous studies which have reported a reduction in TH1 responses to be associated with poor outcomes in TB patients [16] and latently infected individuals [17] with concurrent helminth infection. Helminth-induced regulatory (Treg) responses such as TGF-β and IL-10 production have also been implicated in *S. mansoni*-induced progression to active TB of HIV-1 infected Ugandans [18]. It was also established that deworming of helminth-infected individuals restores cellular immune responses to mycobacterial purified protein derivatives (PPD) [19-21]. Similarly, deworming of helminth-infected Ethiopians immigrants in Israel resulted in increased cellular immune responses against HIV- and *M. tb*-specific antigens compared to untreated individuals [22], suggesting deteriorating immune responses and poor clinical outcomes in helminth-infected individuals might not be a result of inadequate nutrition or sanitation. Several reports have also indicated helminth-mediated modulation of vaccine responses. Children with prenatal sensitization to filariae and schistosomes were reported to display a down-regulation in TH1 responsiveness to BCG vaccination [23] and animal co-infection models have further demonstrated that a pre-existing infection with a lung-migrating helminth, can inhibit development of protective innate anti-TB responses by inducing the IL-4 receptor pathway and accumulation of alternatively activated macrophages [24]. In summary, most reports indicate that helminth infection significantly affects TB susceptibility. In contrast, very little data addressing the effect of mycobacterial infection on host immunity to helminth infections are available.

In the current study, we assessed the influence of co-infection on immune responses against the individual pathogens. We established a BALB/c co-infection model using *Mycobacterium bovis* (*M. bovis*) BCG and the gastrointestinal tract-restricted rodent helminth, *Trichuris muris* (*T. muris*) as TH1 and TH2 pathogenic assaults, respectively. The *M. bovis* BCG murine infection model is routinely used for studying anti-mycobacterial responses during latency as the associated immune response is similar to that induced during human *M. tb* infection [25], whereas *T. muris* infection serves as a well described model for gastrointestinal tract restricted human soil-transmitted helminth (STH) infection [26]. We explored the possibility that concurrent infection with two pathogens, normally cleared by mice during single pathogen infection, might lead to mutually inhibitory immune dynamics and subsequent uncontrolled infection.

## Methods

### Animals

Specified pathogen free (SPF) female BALB/c mice (WT and IL-4 knock-out strains) between 6–8 weeks of age, were kept at the Faculty of Medicine and Health Sciences Animal Unit, Stellenbosch University (SU; South Africa) under conditions compatible with the SU guidelines for the care of animals. All procedures were approved by the SU Animal Ethics Board [Project license: 2003/186/p].

### Parasite enumeration and antigen preparation

*T. muris* eggs were donated by Allison Bancroft (University of Manchester, UK). Egg propagation in BALB/c IL-4 knock-out mice (gift from Frank Brombacher, University of Cape Town, South Africa), helminth collection, and excretory/secretory (E/S) antigen preparations, were performed as described previously [27,28]. Helminth burdens were determined by quantification of intestinal adult worms by examining faecal matter under a dissection microscope. *Mycobacterium bovis* BCG Pasteur (donated by Robin Warren, SU, South Africa) was propagated to logarithmic growth phase in Middlebrook 7H9 (Difco) liquid culture, supplemented with 0.2% glycerol, 0.05% Tween 80 and 10% albumin-dextrose-catalase (ADC, Merck) at 37°C. Bacterial proliferation was assessed by manual counting of colony forming units (CFU) from serial dilutions of homogenized lungs and spleens, plated on Middelbrook 7H11 (Difco) agar plates supplemented with 0.2% glycerol and 10% oleic acid-albumin-dextrose-catalase (OADC, BD Biosciences).

### Co-infection protocol

**Figure 1:**
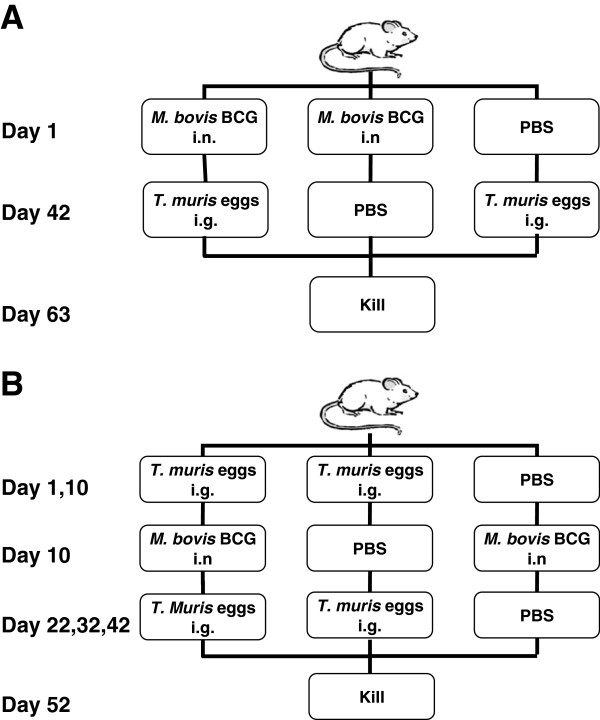
**Experimental design. (A)** BALB/c mice were infected i.n. with *M. bovis* BCG on day 1, followed by i.g. *T. muris* infection on day 42. Mice were killed on day 63 and the relevant tissues collected for further analysis. **(B)** BALB/c mice were infected i.g. with *T. muris* every 10 days starting on day 1. Animals were co-infected i.n. with *M. bovis* BCG on day 10. Mice were killed on day 52 and the relevant tissues collected. Appropriate single infections and PBS controls were included in parallel for both protocols. Experiments were performed with 5 to 10 animals per group. P values <0.05 were considered statistically significant. (ns = non significant).

Two infection protocols were used during this study. Each experiment consisted of 3 groups of 5–10 animals per group. Groups included *M. bovis* BCG-*T. muris* co-infected, BCG-only infected and *T. muris-*only infected mice. The first protocol (Figure 1A) was intended to establish a chronic, low grade *M. bovis* BCG infection that was subsequently followed by a TH2-inducing *T. muris* infection. Mice were infected intranasally (i.n.) with 1–5 × 10^5^ CFU BCG bacilli per mouse or an equal volume of PBS. Briefly, mice were lightly anesthetized by intraperitoneal (i.p.) injection of a 200 μl mixture consisting of Ketamine (12 mg/ml Anaket-V, Centaur Labs) and Xylazine (1.6 mg/ml, Rompun, Bayer). Mice were gently lifted by the loose skin at the throat, and kept upright with its head tilted back and the nose pointed up. Using a pipette with a sterile tip, 40 μl of the declumped mycobacterial suspension was applied to the nostrils. Animals were maintained upright for another 30 seconds to ensure complete delivery to the respiratory system. Six weeks (day 42) later, mice were infected under light anaesthesia intragastrically (i.g.) with 200–250 (low dose) or 500–600 (high dose) embryonated *T. muris* eggs or an equal volume of PBS. At week 9 (day 63), mice were culled and the relevant organs removed for investigation. The second protocol (Figure 1B) was designed to first establish a TH2-inducing *T. muris* infection prior to challenge with *M. bovis* BCG infection. Animals were infected i.g. with 200–250 embryonated *T. muris* eggs or an equal amount of PBS on day 1 and every 10 days thereafter until experimental completion. On day 10, animals were infected i.n. with 1–5 × 10^5^ CFU BCG bacilli or an equal volume of PBS. After 6 weeks (day 52), all mice were humanely euthanized and the relevant organs removed for investigation. Experiments were completed in triplicate at three separate times.

### Immune phenotyping and intracellular cytokine analysis

Immune phenotyping was performed using single cell suspensions from spleens and mesenteric lymph nodes (MLNs). Intracellular cytokine expression was determined following stimulation with 50 ng/ml Phorbol 12-myristate 13-acetate (PMA) (Sigma), 1 μg/ml Ionomycin (Sigma) and 10 μg/ml Brefeldin A (BFA) (Sigma) for 4 hours at 37°C and 5% CO_2_. Cells were resuspended in PBS containing 1% BSA and 0.1% Sodium Azide (wash buffer) and stained for 30 minutes with fluorochrome conjugated anti-mouse antibodies against CD3, CD4, CD8, CD25, B220, Foxp3, IFN-γ and IL-4 (BD Biosciences, Caltag or Biolegend). Cells were fixed with 1% formaldehyde, washed and resuspended in wash buffer. Lymphocyte populations were determined based on their Forward/Side scatter profile and gates set with the help of appropriate FMOs and Isotype controls. Acquisitions were performed on a FACSCalibur (BD Biosciences) using appropriate instrument settings, color compensation and isotype controls for all antibodies. At least 5 × 10^4^ lymphocyte events were acquired and data analysis performed using CellQuest software (BD Bioscience).

### *In vitro* pathogen-specific cytokine analysis

Spleen (1 × 10^7^ cells/ml) single cell suspensions were stimulated for 24 hours with live BCG cultures (MOI 5:1), 50 μg/ml E/S antigen or culture media as control at 37°C, 5% CO_2_. Culture supernatants were used for cytokine concentration analyses using the luminex bead-array technology (LINCO Research) to test for the soluble cytokines IFN-γ, TNF-α, IL-4, IL-10, IL-13 and IL-17 using a Bio-Plex platform (Bio-Rad Laboratories). Background readings were controlled by subtraction of unstimulated control sample measurements. Values were checked against internal quality controls to monitor analysis accuracy within specified concentration ranges.

### Nucleic acid extraction and relative quantitative real time PCR

Total RNA was extracted from the upper right lobe of mouse lungs and spleen tips using Trizol (Gibco BRL) and subsequently treated with a DNA-free kit (Ambion) to remove contaminating DNA. First strand cDNA was transcribed using the QuantiTect Reverse Transcription kit (Qiagen) according to the manufacturer’s protocols. Relative quantification of IFN-γ, IL-4, IL-10, TGF-β and Foxp3 were performed using SYBR Green PCR Master Mix kit (Roche), cDNA (500 μg) and primers (0.5 μM) on the LightCycler system v3.5 (Roche). All primers were designed to span intron-exon boundaries (Table 1). The delta-delta Ct method was used to calculate relative gene expression levels between two samples. Gene expression was assayed quantitatively and normalized to that of a housekeeping gene (GAPDH, HPRT, 18S-RNA) to obtain a RNA ratio in order to establish the relevant change in RNA expression [29].

**Table 1:** List of primer sequences used for relative quantitative real-time PCR

**Target**	**Forward**	**Reverse**
HPRT	GACTGTAGATTTTATCAGACT	GTCTGGCCTGTATCCAACACTTC
GPDH	GGTGGCAGAGGCCTTTG	TGCCGATTTAGCATCTCCTT
^*^18S [30]	GTCTGTGATGCCCTTAGATG	AGCTTATGACCCGCACTTAC
^*^TGF-β [30]	CCGCAACAACGCCATCTATG	CTCTGCACGGGACAGCAAT
^*^IFN-γ [31]	AAGTTCTGGGCTTCTCCTCCTG	GCCAGTTCCTCCAGATATCCAAGA
^*^IL-10 [30]	CTGGACAACATACTGCTAACCG	GGGCATCACTTCTACCAGGTAA
^*^IL-4 [31]	TCAACCCCCAGCTAGTTGTC	TTCAAGCATGGAGTTTTCCC
GATA3	CTGGAGGAGGAACGCTAATG	GGTTGAAGGAGCTGCTCTTG
Tbet	AGCAAGGACGGCGAATGTT	GGGTGGACATATAAGCGGTTC
^*^Foxp3 [30]	CACAATATGCGACCCCCTTTC	AACATGCGAGTAAACCAATGGTA

^*^Primer sequences adapted from reference.

### Histology

Left upper lung lobes were fixed in 10% buffered formalin, embedded in paraffin blocks and sections (3-5 μm) stained with Haematoxylin and Eosin (H&E) for light microscopy. Pulmonary histopathological scoring was performed in a blinded fashion and calculated separately for each lung section as previously described [32]. In brief, a scale of 0 to 4 was used to individually score the level of peribronchiolitis, perivasculitis, interstitial pneumonitis and alveolitis of each section in order to obtain an average score for each lung. A score of 0 was based upon observation of normal, uninfected mouse lung samples and a score of 4 on previous studies of greatest inflammatory change and pathology brought about by i.n *M. bovis* BCG infection in BALB/c mice. Scoring of gastrointestinal histopathology was achieved by measuring mucus production, presence of mast cells and mitotic body enumeration in fixed caecum tips imbedded in paraffin blocks. Sections (3-5 μm) were used for Periodic Acid Schiff (PAS) staining to score goblet cell-mucus production within caecal crypts as the percentage PAS positive stain in the crypt epithelium and lamina propria. Acidified toluidine blue staining was used for the quantification of mast cells in caecum tip samples and enumeration of mitotic bodies within caecum crypts. Scoring was conducted from two sets (cross sectional and longitudinal) of 20 caecal crypt units per animal. All slides were evaluated using the ZS300 Imaging system v.3.0 (Carl Zeiss Vision).

### Statistical analysis

Data was analyzed using STATISTCA v.7 (StatSoft) software. Nonparametric analysis and Mann–Whitney U tests were performed for comparison between groups and the data presented as median values. Multiple group analysis included the multiple comparison correction (Bonferroni). Statistically significant differences were judged as p ≤ 0.05.

## Results

### *M. bovis* BCG clearance and lung pathology is not influenced by an established or successive *T. muris* infection

**Figure 2:**
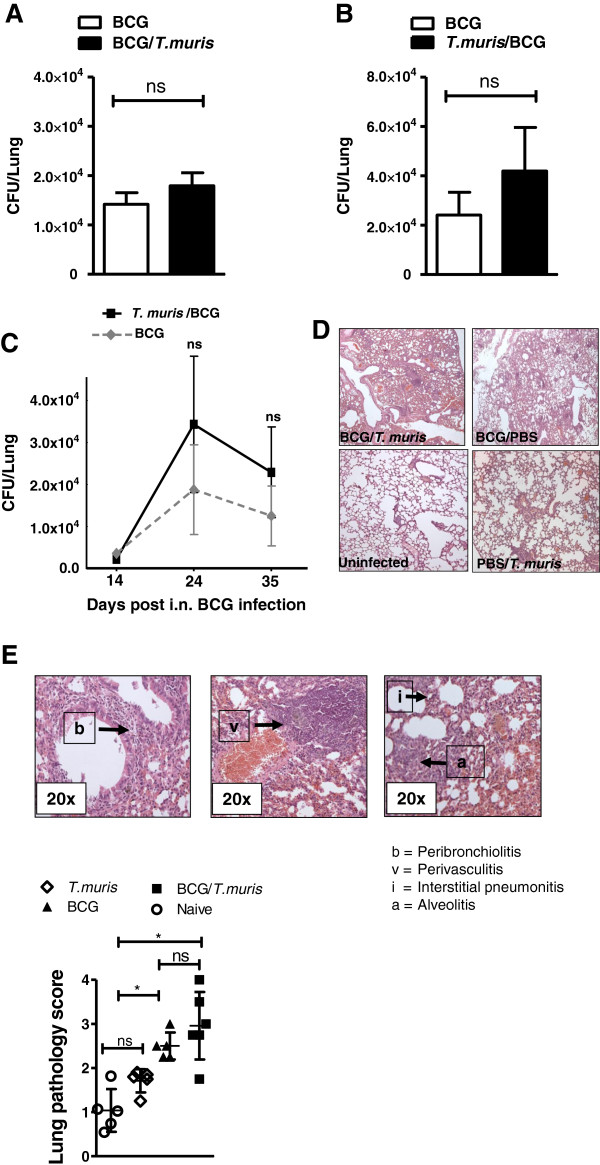
***M. bovis*BCG clearance and mycobacterial-induced lung pathology is not influenced by an established or successive*T. muris*infection. (A)** Viable pulmonary *M. bovis* BCG CFU numbers at experimental endpoint in co-infected (black) and BCG-only (clear) infected BALB/c mice infected according to experimental design as shown in Figure 1A. Data display mean ± SEM, representing 3 individual experiments of 5–6 animals per group. **(B)** Viable pulmonary *M. bovis* BCG CFU numbers at experimental endpoint in co-infected (black) and BCG-only (clear) infected BALB/c mice infected according to experimental design as shown in Figure 1B. Data display mean ± SEM, representing 3 individual experiments of 5–6 animals per group. **(C)** Viable pulmonary *M. bovis* BCG CFU growth curve data of co-infected (black) and BCG-only (clear) infected mice at days 14, 24 and 35 post BCG infection **(D)** Representative histological H&E stained lung sections captured at 10x magnification illustrating the differences in histopathology between BCG/*T.muris* co-infected, BCG-only infected, uninfected and *T. muris*-only infected BALB/c mice infected according to experimental design as shown in Figure 1A. **(E)** Pulmonary histopathological scoring was performed in a blinded fashion according to the degree of peribronchiolitis (b), perivasculitis (v), interstitial pneumonitis (i) and alveolitis (a) per lung. Average pulmonary scores of BALB/c mice infected according to experimental design as shown in Figure 1A. Groups included naive (circle), *T. muris*-only (diamond), BCG-only (triangle) and co-infected (square) mice. Data display mean ± SD, representing 2 individual experiments of 5–6 animals per group. P values <0.05 were considered statistically significant. (*p ≤ 0.05, ns = non significant).

The influence of *T. muris* infection on host ability to control a chronic, low grade *M. bovis* BCG infection in BALB/c mice was investigated for both experimental protocols (Figure 1A and B). Results demonstrated that an ongoing helminth-induced TH2 immune background, pre-established by *T. muris* trickle infection, failed to alter mycobacterial proliferation and dissemination when compared to *M. bovis* BCG-only infected mice in the lungs (Figure 2A) and spleen (data not shown). Similarly, initiation of a TH2 immune environment subsequent to BCG infection, resulted in equivalent pulmonary bacterial burdens between co-infected and BCG-only infected groups (Figure 2B). These end point CFU findings were confirmed by growth curve data demonstrating no significant difference in pulmonary mycobacterial burden between co-infected and *M. bovis* BCG-only infected mice at several time points post *M. bovis* BCG infection (Figure 2C). Histological scoring of both infection protocols indicated that *T. muris*-only infected mice displayed normal lung pathology with only minimal cell infiltration compared to naive mice, whereas the degree of pulmonary pathology and the cellular composition and organization in the lungs following *M. bovis* BCG co-infection were significantly increased (Figure 2D and E). No significant differences in pulmonary inflammatory scores could be detected between BCG-only and co-infected mice for either infection protocols (Figure 2D, 2E and Additional file 1: Figure S1).

### Previously established BCG infection delays *T. muris* expulsion in co-infected animals

**Figure 3:**
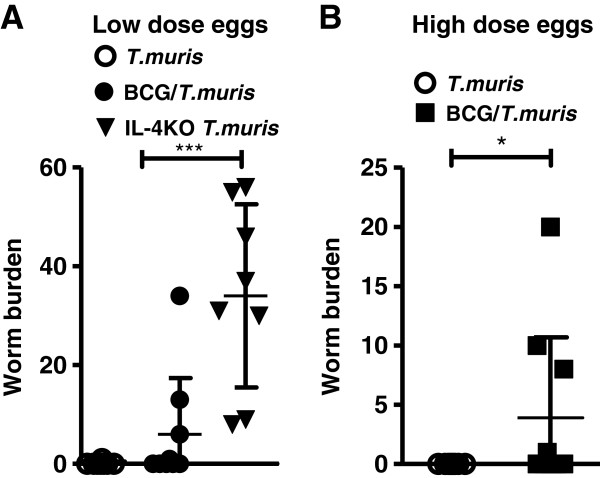
**Co-infection increases retention of*T. muris*helminths.** The burden of *T. muris* worms were determined from the caecum and 3 inches of the colon of BALB/c mice infected according to the experimental design as shown in Figure 1A. Worm counts in *T. muris*-only BALB/c (clear circle) and IL-4KO (triangle) strains and co-infected BALB/c (square) mice infected with a low **(A)** and high **(B)** dose of helminth eggs. Data represents combined results of 2 individual experiments of 4–5 animals per group. P values <0.05 were considered statistically significant. (*p ≤ 0.05).

The influence of *M. bovis* BCG co-infection on eradication of *T. muris* in BALB/c mice was evaluated as worm expulsion for both experimental protocols (Figure 1A and B). In each case, susceptible IL-4KO mice with disrupted protective TH2 responses, were included as controls of delayed worm clearance [33]. Following the infection strategy in Figure 1A, the helminth burden at experimental completion demonstrated that almost half (44%; 4/9) of mice with an established chronic BCG infection, that were subsequently co-infected with a low dose of helminth eggs, still presented with *T. muris*, whereas significantly more animals (88%; 7/8) from the *T. muris*-only infected group had cleared all helminths (Figure 3A). Both groups displayed significantly lower worm burdens compared to IL-4KO mice infected with *T. muris* only (Figure 3A). Similar results were observed in experimental repeats using a high dose of helminth eggs, showing helminth clearance in (100%; 0/9) *T. muris*-only infected BALB/c mice, whereas *T. muris* expulsion failed in (40%; 4/10) *M. bovis* BCG co-infected BALB/c mice (Figure 3B). However, when the infection sequence was reversed, where an initial *T. muris* infection was followed by a subsequent BCG infection (Figure 1B), repeat experiments consistently indicated helminth clearance in >90% of both co-infected and *T. muris*-only infected mice (data not shown).

### Co-infection exacerbates cell proliferation in caecum tips

**Figure 4:**
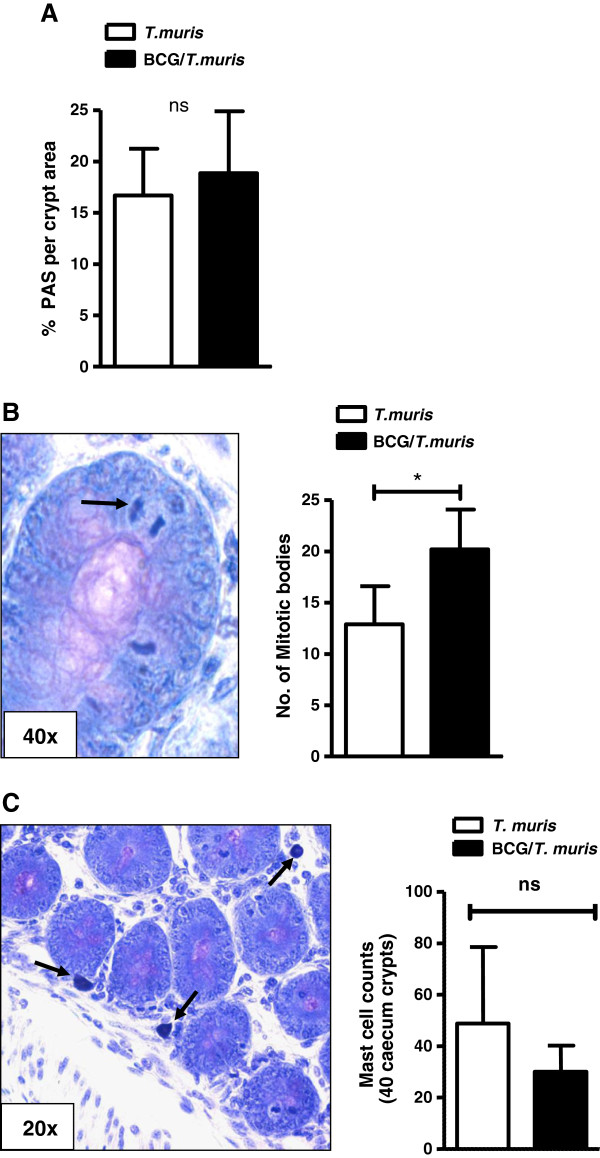
**Co-infection increases mitotic figures in the caecum crypts. (A)** Histological analysis of goblet cell numbers as determined by the percentage PAS^+^ cells (indicated by arrow) per 2 x 20 cross sectional crypt units in *T. muris*-only (clear) and co-infected (black) BALB/c mice infected according to the experimental design as shown in Figure 1A. Data display median ± min-max, representing 2–3 individual experiments of 5 animals per group. **(B)** Toluidine blue stained mitotic bodies (indicated by the arrows) were counted in 2 x 20 crypts/slide. Numbers of mitotic bodies as determined from cross-sectional and longitudinal crypt units in co-infected (black) and *T. muris*-only (clear) infected BALB/c mice infected according to Figure 1A. Data display median ± min-max, representing 2–3 individual experiments of 5 animals per group **(C)** Toluidine blue staining for the assessment of mast cells (indicated by arrows) in cross sectional and longitudinal crypt units demonstrated few mast cells within the lamina propria and crypt epithelium of the caecum tissue with most mast cells residing within the submucosa surrounding the caecum. Bar graph indicating the numbers of mast cells measured in co-infected (black) and *T. muris*-only (clear) infected groups per 40 caecum crypts. Data display median ± SD of 5 animals per group. P values <0.05 were considered statistically significant. (ns = non significant).

A striking observation was the massive amount of mucus present in the caeca and colons of mice co-infected according to either experimental protocol (Figure 1A and B) in comparison to *T. muris*-only infected mice. Although PAS stained samples failed to demonstrate significant differences in goblet cell formation or caecal crypt-mucus production between co-infected and *T. muris*-only infected mice (Figure 4A), acidified toluidine blue staining showed significantly increased numbers of mitotic figures in caecum crypts of co-infected animals as identified by their dense chromatic structure (Figure 4B). Very few mast cells were observed within the epithelium or lamina propria of the crypt units in co-infected mice and no significant statistical differences in mast cell recruitment were observed between infection groups (Figure 4C).

### Co-infection increases CD4^+^ splenocyte frequencies and modifies the TH1/TH2 immune balance

**Figure 5:**
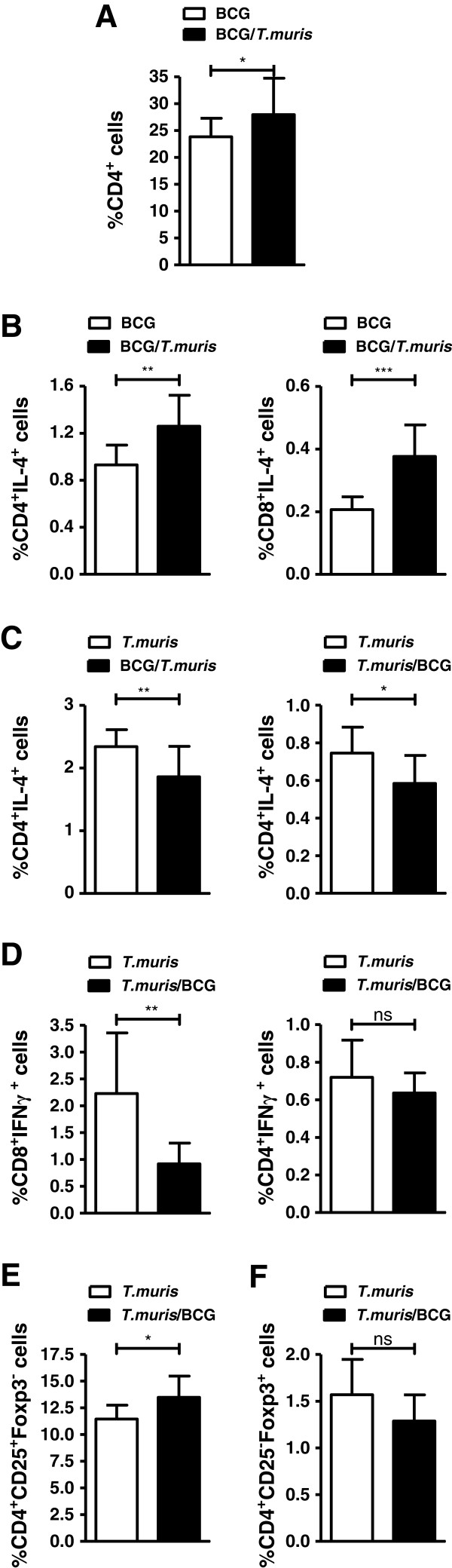
**Co-infection affects the frequency of CD4**^**+**^**and Treg lymphocyte populations and alters*ex vivo*TH1/TH2 cell populations. (A)** Percentages of CD4^+^ splenocytes in BCG-only (clear) and co-infected (black) BALB/c mice infected according to experimental design in Figure 1A. Data display median ± min-max, representing 2–3 individual experiments of 20 animals per group. **(B)** Percentages of IL-4 producing CD4^+^ and CD8^+^ splenocytes in BCG-only (clear) and co-infected (black) BALB/c mice infected according to the protocol in Figure 1B. Data display median ± min-max, representing 2–3 individual experiments of 8–10 animals per group. **(C-D)** Percentages of CD4^+^IL-4^+^, CD8^+^IL-4^+^ and CD4^+^IFN-γ^+^ MLN cell populations in *T. muris*-only (clear) and co-infected (black) BALB/c mice infected according to experimental design in Figure 1B. Data represents experiments with 8–10 animals per group. Percentages of **(E)** activated T cells (CD4^+^CD25^+^Foxp3^-^) and **(F)** inducible regulatory T cells (iTreg) (CD4^+^CD25^-^Foxp3^+^) in MLNs of *T. muris*-only (clear) and co-infected (black) BALB/c mice infected according to experimental design in Figure 1B. Data display median ± min-max, representing 2–3 individual experiments of 8–10 animals per group. P values <0.05 were considered statistically significant. (*p ≤ 0.05, **p ≤ 0.01, ns = non-significant).

Flow cytometric analysis demonstrated that co-infection according to either infection protocol (Figure 1A and B) did not impact lymphocyte composition in the spleen or MLN, since no significant differences between infection groups were observed for populations of CD3^+^ T cells or B220^+^ B cells (data not shown). However, analysis of *ex-vivo* lymphocyte subpopulations in BALB/c mice infected according to Figure 1A, revealed an increase in CD4^+^ T helper cell population in the spleens of mice co-infected according to the protocol in Figure 1A, when compared to BCG-only infected mice (Figure 5A). Although no differences in the percentages of natural regulatory T cell (CD4^+^CD25^+^Foxp3^+^) populations were observed between infection groups in either the spleen or MLN (data not shown), co-infection significantly increased the percentage of IL-4-producing CD4^+^ and CD8^+^ splenocytes in comparison to *M. bovis* BCG-only infected controls (Figure 5B). IL-4-producing CD4^+^ and CD8^+^ MLN cells from co-infected mice were however significantly reduced in comparison to *T. muris*-only infected mice (Figure 5C). A marked decrease in CD8^+^IFNγ^+^ MLN cells was also observed in co-infected mice in comparison to mice infected only with *T. muris*, whereas frequencies of CD4^+^ IFNγ^+^ MLN cells were measured at similar levels between co-infected and *T.muris*-only infected mice (Figure 5D).

When the infection order was reversed during trickle infection to address the effect of introduction of co-infection with *M. bovis* BCG into an established helminth-induced TH2 environment (Figure 1B), a significant increase in activated effector T cell (CD4^+^CD25^+^Foxp3^-^) percentages in MLNs of co-infected animals was observed in comparison to *T. muris*-only infected controls (Figure 5E). A trend towards decreased frequencies of inducible regulatory T cells (iTreg) (CD4^+^CD25^-^Foxp3^+^) was also observed in the MLNs of co-infected compared to *T. muris*-only infected mice (Figure 5F). No significant differences in *ex vivo* cytokine production between infection groups were observed for CD4^+^ and CD8^+^ lymphocytes in the spleen or MLNs (data not shown).

### Co-infection reduces pathogen-specific TH1 and TH2 immune responses

Pathogen-specific TH1/TH2/TH17/Treg cytokine immune responses in the spleen were analyzed only in BALB/c mice infected according to the protocol in Figure 1A, since no significant differences in *ex vivo* T cell cytokine production between infection groups were observed in the spleens or lungs of mice infected according to the protocol in Figure 1B.

**Figure 6:**
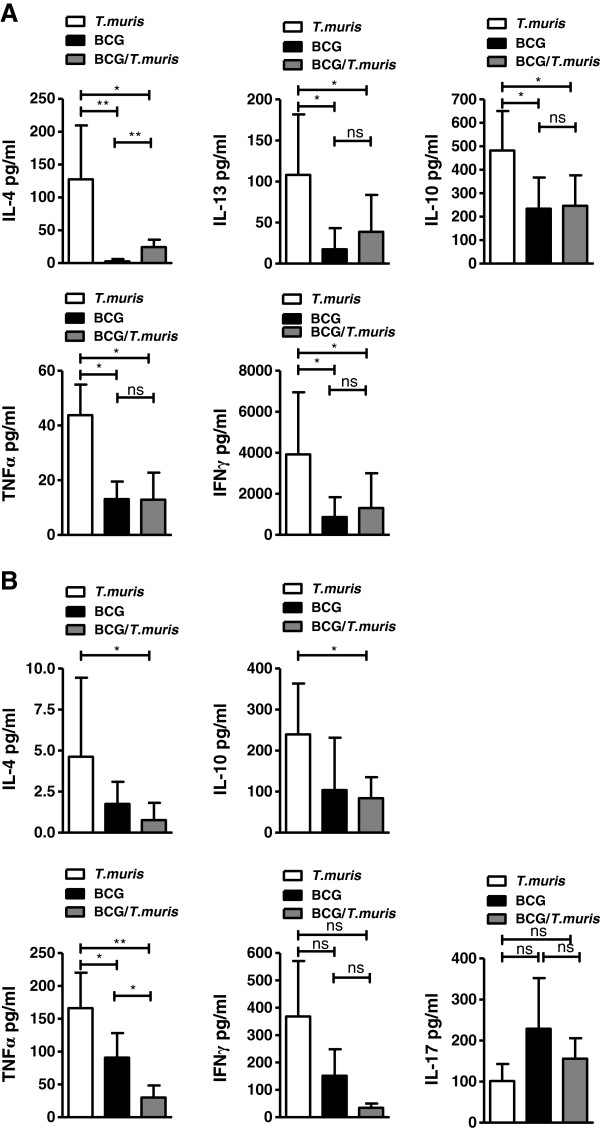
**Co-infection leads to altered pathogen-specific TH1 and TH2 immune responses.** TH1 and TH2 cytokine concentrations were measured from 24 hour **(A)** E/S stimulated and **(B)** BCG-stimulated splenocyte cultures of co-infected (grey), *T. muris*-only (clear) and BCG-only (black) BALB/c mice infected according to the protocol illustrated in Figure 1A. Results from stimulated values were corrected for background unstimulated controls. Data display median ± min-max, representing 2–3 individual experiments of 5 animals per group. P values <0.05 were considered statistically significant. (*p ≤ 0.05, **p ≤ 0.01, ns = non-significant).

E/S stimulated splenocytes from both co-infected and BCG-only infected mice displayed a prominent reduction in TH2/Treg (IL-4, IL-13 and IL-10) cytokine production when compared to *T. muris*-only infected animals, although IL-4 levels were significantly increased in co-infected compared to BCG-only infected mice (Figure 6A). Similarly, E/S-specific TH1 cytokines (TNF-α and IFN-γ) were reduced in both the co-infected and BCG-only infected groups with respect to *T. muris*-only infected animals (Figure 6A). No notable differences between the infection groups were observed for helminth-specific IL-17 production (data not shown).

BCG-stimulated splenocytes displayed notably low concentrations of TH2 (IL-4 and IL-13) cytokines in all infection groups. Although no significant differences in concentrations of the cytokines, IFN-γ and IL-17 (Figure 6B) were measured between infection groups, co-infection significantly decreased production of the cytokines TNF-α, IL-10 and IL-4 in comparison to *T. muris*-only and/or BCG-only infected mice (Figure 6B).

### Co-infection reduces the pulmonary cytokine gene expression profile relative to BCG-only infected animals

**Figure 7:**
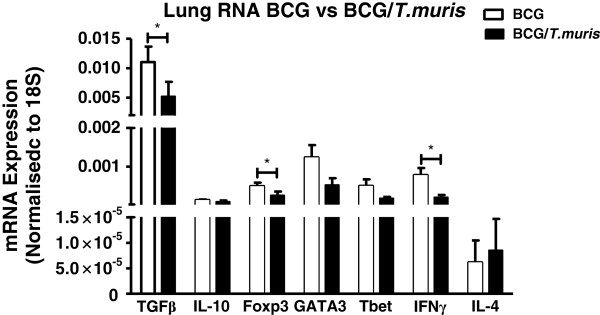
**Co-infection decreases the expression ratio of pulmonary RNA cytokine transcripts relative to those of BCG-only infected BALB/c mice.** BALB/c mice were co-infected (black) according to the protocol illustrated in Figure 1A with BCG-only (clear) infected mice included as controls. At week 9, total RNA was extracted from the right upper lung lobe, cDNA produced and the relative gene expression ratio in co-infected mice relative to that of BCG-only infected mice, determined by real-time PCR. Following HKG normalization and delta-delta Ct analysis, the expression ratio of the genes TGF-β, IL-10, Foxp3, GATA3, T-bet, IFN-γ were calculated. Data display median ± SE, representing 8–10 animals per group. P values <0.05 were considered statistically significant in comparison to BCG-only infected. (*= p < 0.05).

To assess whether the immunological changes observed in mice infected according to the infection protocol indicated in Figure 1A, also extends to alterations in pulmonary and splenic gene expression levels, the relative gene expression of co-infected mice and BCG-only infected mice was determined. At week 9, the relative gene expression ratios from co-infected mice demonstrated significantly decreased RNA levels in the lungs for TGF-β (p = 0.034), Foxp3 (p = 0.042) and IFN-γ (p = 0.012) relative to BCG-only infected mice (Figure 7). The levels of IL-10 (p = 0.072) also showed a trend towards decreased expression across these two groups (Figure 7). Analysis of RNA profiles in the spleen failed to show significant variations in expression levels for any of the genes measured, between co-infected and BCG-only infected groups (data not shown).

## Discussion

In this study, we demonstrate the capability of the gastrointestinal tract restricted helminth, *T. muris*, to induce local and systemic TH2 immune responses that affect immunity to *M. bovis* BCG. Of particular interest was the significant reduction in BCG-specific TNF-α and IL-10 cytokine concentrations and significant increase in IL-4-producing CD4^+^ and CD8^+^ T cells in the spleens of co-infected mice, in comparison to BCG-only infected mice. In addition, we show that co-infection significantly reduced pulmonary IFN-γ, TGF-β and Foxp3 gene expression, relative to BCG-only infected mice. Collectively, our data show a down-regulation in pulmonary TH1 and Treg-associated responses and the induction of systemic TH2 responsiveness following co-infection. Nevertheless, lung and systemic bacterial burdens remained unaffected in co-infected mice and did not translate into alterations in pulmonary histopathology with respect to BCG-only infected mice, suggesting that protective host immune responses could be sufficiently compartmentalized to appropriately respond to the mycobacterial infection. Previous reports have demonstrated the host’s ability to fully compartmentalize immunity during co-infection with TH1 and TH2-inducing pathogens at different sites of the mammalian body [34]. While helminth co-infection has been shown to negatively influence host control of other intracellular pathogens, several reports suggest that this outcome is specific to the helminth species investigated [35-38]. Even so, *T. muris* infection marginally increased pulmonary cellular infiltration with respect to naive mice, likely due to systemic inflammation caused by the helminth infection or the presence of helminth antigens. Although not discussed here, work done by us shows that neither adoptive transfer of splenocytes or MLN leukocytes from helminth-only infected animals, or abrogation of IL-4 in IL-4 deficient mice, resulted in altered mycobacterial burden (unpublished data). These transfer experiments could however not exclude a role for suppressive MLN or spleen cell subsets since purified populations were not used in these experiments. Also, the timing of transfer and the absence of continual pathogen-derived antigen stimulation in the recipient host could play a role in the effector responses and activation status of these cells.

Interestingly, our results show that prior pulmonary *M. bovis* BCG infection also significantly affected local and systemic protective host immune responses to a subsequent *T. muris* infection. Although the lack of *ex vivo* phenotyping data from BCG-only infected mice is a weakness in this infection protocol, co-infected mice displayed a significant reduction in E/S-specific TH1 and TH2 cytokine responses in the spleen, and significantly reduced IL-4 producing CD4^+^ and CD8^+^ T cells and IFN-γ-producing CD8^+^ T cells in the mesenteric lymph nodes when compared to *T. muris*-only infected mice. In support of a functional role for this reduction in *T. muris*-specific immunity, we demonstrated an associated delay in helminth clearance and increased helminth-related intestinal pathology in co-infected mice, when compared to *T. muris*-only infected mice. These intestinal pathological changes were characterized by increased cell turnover, suggesting increased apoptosis or cell damage, necessitating cell replacement [39]. Intestinal crypt cell apoptosis was previously reported to occur following *T. muris* infection and subsequently shown to be reduced following neutralization of IFN-γ and TNF-α [40]. In parallel with this we observed an increase in intestinal mucus production, which likely operates as a compensatory mechanism to aide expulsion of persisting parasites. Our results verify reports illustrating that *M. bovis* co-infection increase helminth parasite burden and correlates with decreased IL-4 and IL-13 cytokine production [41]. Our findings also agree with early reports demonstrating a reduction in protective immune responses and a delay in *T. muris* expulsion during other co-infections with *Nematospiroides dubius*, *Plasmodium berghei* or *Trypanosoma brucei*[42-44]. It is well established that resolution of *T. muris* infection is characterized by the production of TH2 cytokines, resulting in intestinal goblet cell hyperplasia and increased intestinal epithelial cell turnover [45,46]. On the other hand, mast cells, γδ T cells and eosinophils are suggested as dispensable for *T. muris* expulsion [45,47] and the contribution of B cells and antibody responses remains controversial [48-50]. Previous reports convincingly show that *T. muris* infection is delayed following depletion of CD4 T cells [51], inhibition/down-regulation of TH2 cytokines [33,45] and increased TH1 polarization [52]. It is therefore likely that our observation of reduced helminth-specific TH2 responses in this co-infection model could, at least in part, explain the delay in *T. muris* expulsion, although induction of TH1 immune responses to *M. bovis* BCG following *T. muris* infection would also influence parasite expulsion. Interestingly, altering the infection sequence to elucidate the effect of a subsequent mycobacterial infection on an established helminth-induced TH2 immune response did not have any negative influence on mycobacterial or helminth clearance by the host. This is most likely to be due to the rapid clearance of the helminth infection and development of resistance to re-infection, or due to the presence of an established TH1 immune response for altering helminth clearance [53].

These modified pathogen-specific and non-specific immune responses following co-infection provide clear evidence that both pathogens have the ability to reciprocally modulate immune responses towards each other at their individual infection foci. More importantly, the down-regulation of overall immune responsiveness in the context of both infections suggests co-infection-induced immune suppression as a possible mechanism. Several reports confirm that chronic immune activation during helminth infections could initiate immune suppression or anergy [22]. Here, we show significant increases in the frequency of systemic CD4^+^ T cells and effector T cells in MLN of co-infected animals, suggesting increased immune activation following co-infection. Although the presence of immune suppressive regulatory cell populations was investigated, no differences in the frequencies of Treg populations could be detected between infection groups in either of the BALB/c co-infection models. As Treg cells exert their suppressive function in a cytokine dependent manner and also interact with other T cells and APC directly, the implications of co-infection on regulatory immune mechanisms are not clear. Changes in IL-10, Foxp3 and TGF-β gene expression reveal that the role of Tregs cannot be excluded. Our results could point towards a role for other immune regulatory cell populations, and current research efforts are focused towards the involvement of innate nuocytes and myeloid derived suppressor cells (MDSCs) [54,55].

## Conclusion

In summary, the work presented here supports the hypothesis that co-infection by two unrelated and anatomically separated pathogens can reciprocally alter the host’s immune response to either infection. Co-infection altered host pathology and the host’s ability to expel invading helminth parasites; however the magnitude of the impact was dependent on the sequence of co-infection. These phenotypic changes were associated with alterations in organ-restricted TH1/TH2/Treg immune balance, immune suppression and pathogen-specific and non-specific cytokine responses. It is likely that multiple mechanisms may operate concurrently and further research is needed to identify the critical factors involved, although our results strongly support a mechanism whereby chronic immune activation leads to hyporesponsiveness resulting in reduced pathogenic control during co-infection. These findings demonstrate the complexity of immune response regulation and systemic interaction between innate and adaptive immunity and thereby hightlights the need for greater understanding of the role of infection history on the evolution of host immunity.

## Competing interests

The authors declare that they have no competing interests.

## Authors’ contributions

Study concept & design – GW, HJN. Acquisition of data – HJN, LK. Statistical analysis – HJN, NDP. Analysis and interpretation of data – GW, HJN, NDP. Drafting of the manuscript – HJN, NDP. Critical revisions to the manuscript – GW, AGL, NDP, PVH. Obtained Funding – GW, HJN. Study Supervision – GW. All authors read and approved the final manuscript.

## Authors’ information

Hendrik J Nel and Nelita du Plessis co-first author.

## Supplementary Material

Additional file 1: Figure S1 Representative histological H & E stained lung sections captured at 10x magnification illustrating the differences in histopathology between *T. muris*/BCG co-infected, BCG-only infected, uninfected and *T. muris* - only infected BALB/c mice infected according to experimental design as shown in Figure 1B. Click here for file
