# Supplementary figures and images for: Mycobacterium bovis BCG infection severely delays Trichuris muris expulsion and co-infection suppresses immune responsiveness to both pathogens
Source: BMC Microbiol. 2014 Jan 17;14:9. doi: 10.1186/1471-2180-14-9 (PMC3898725; doi:10.1186/1471-2180-14-9)

S1)

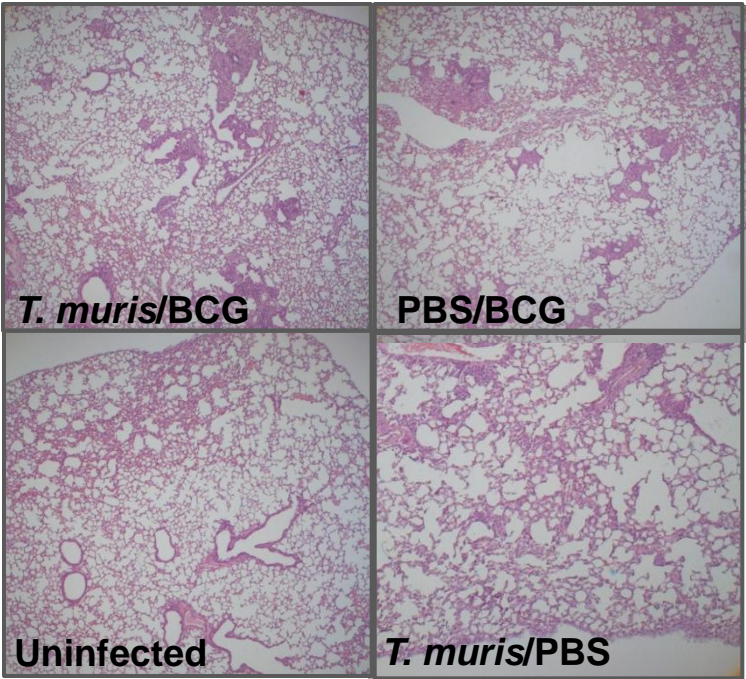

Figure S1

Supplement: Additional file 1: Figure S1 — Representative histological H & E stained lung sections captured at 10x magnification illustrating the differences in histopathology between T. muris/BCG co-infected, BCG-only infected, uninfected and T. muris - only infected BALB/c mice infected according to experimental design as shown in Figure 1B. [file 1471-2180-14-9-S1.pdf]
